# Supplementary material for: Multivalent contacts of the Hsp70 Ssb contribute to its architecture on ribosomes and nascent chain interaction
Source: Nat Commun. 2016 Dec 5;7:13695. doi: 10.1038/ncomms13695 (PMC5150220; doi:10.1038/ncomms13695)
Supplement: Supplementary Information — Supplementary Figures [file ncomms13695-s1.pdf]

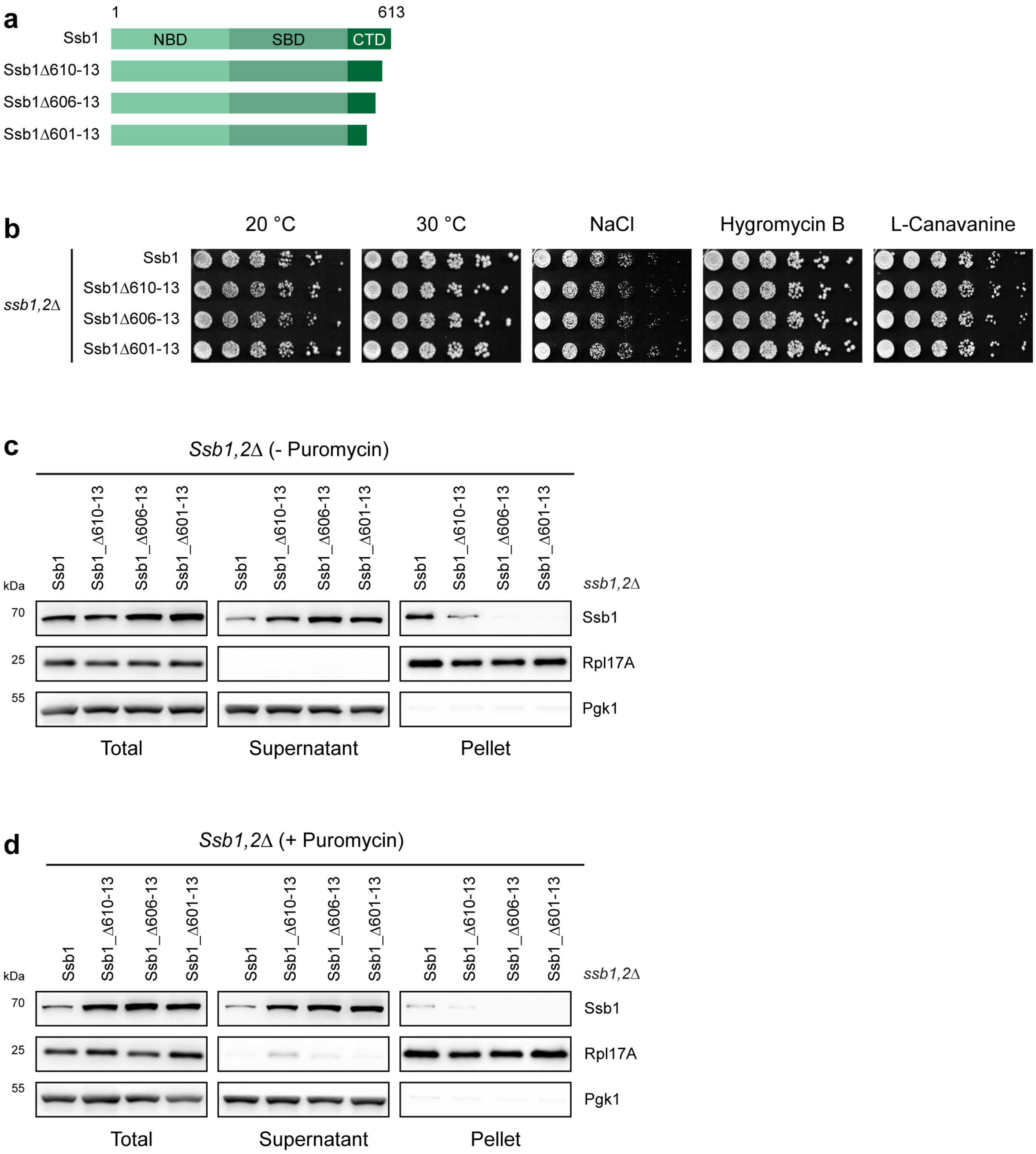

Supplementary Figure 1: Stepwise deletion of the C-terminus of Ssb1 gradually reduces ribosome binding. a) Schematic overview of Ssb domains and the constructs used lacking 4, 8 or 13 C-terminal residues. **b)** Growth analysis of different Ssb1 mutants with gradually shortened C-terminus. *Ssb1,2Δ* cells transformed with different Ssb1 versions were grown to exponential phase, adjusted to OD_{600} = 0.4 and spotted in fivefold serial dilutions onto SC-URA plates (that may contain certain additives) which were incubated at 30 °C for two days or at 20 °C for five days. **c)** Ribosome sedimentation assay to investigate ribosome binding of different Ssb1 mutants. Cells were grown to exponential growth phase, lysates were adjusted to same A_{260} levels and 1.5 A_{260} units were loaded onto a 25 % (w/v) sucrose cushion followed by ultracentrifugation. Equal amounts of adjusted total, supernatant and ribosomal pellet fractions were analyzed via SDS-PAGE followed by Western blotting. **d)** Ribosome sedimentation as shown in c) including 1 mM puromycin to release the nascent chain.

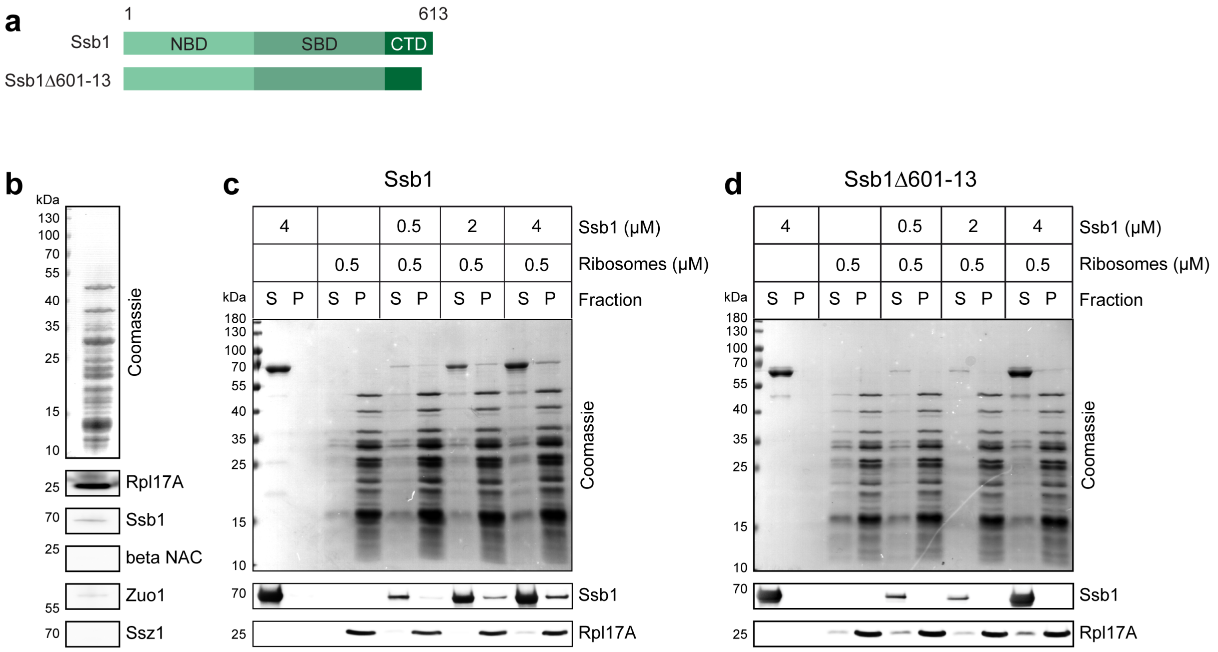

Supplementary Figure 2: Deletion of Ssb1 residues 601-13 abolishes ribosome binding *in vitro*. a) Schematic overview of Ssb domains and the constructs used. Ssb1Δ601-13 lacks 13 C-terminal residues. **b)** Salt-stripped and puromycin treated yeast ribosomes were tested for the presence of ribosome-associated factors by either SDS-PAGE and Coomassie staining (top) or by immunological detection of different proteins (bottom). **c)** Add back of wt Ssb1 to purified yeast ribosomes. 0.5 μM puromycin and high salt-stripped yeast ribosomes were incubated with different concentrations of wt Ssb1 protein (0.5, 2 and 4 μM). Ribosomes were sedimented and the post-ribosomal supernatant (S) as well as the ribosomal pellet (P) was further analyzed by SDS-PAGE and Coomassie staining (top) or by immunological detection of ribosomal Rpl17A and Ssb protein (bottom). **d)** Add back of truncated Ssb1Δ601-13 protein to purified yeast ribosomes as described in c).

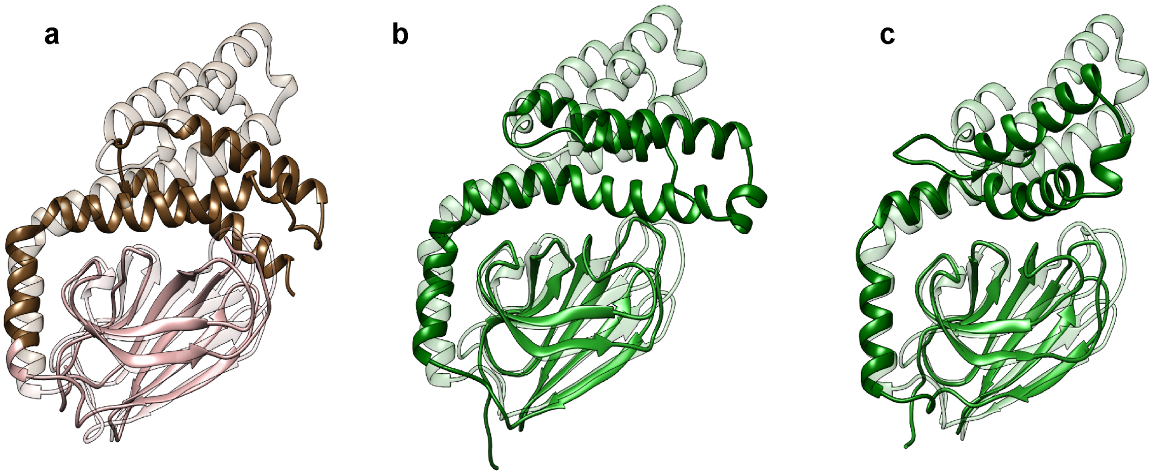

Supplementary Figure 3: Deletion of the 13 C-terminal residues affects the structural stability of the CTD of Ssb. Superposition of starting (transparent color) and final (solid color) structures after 750 ns of MD (molecular dynamics) simulations of Hsp70 chaperone SBD (substrate binding domain; middle color) and CTD (C-terminal domain; dark color). **a)** Simulation of Ssa, which shows interaction between the SBD and CTD and a lid closing movement of the CTD. **b)** For wild type Ssb1 the CTD remains intact and closes in on the SBD. **c)** Simulation of the Ssb1Δ601-13 mutant as shown in b). The CTD unfolds from the terminus and the long helix bends.

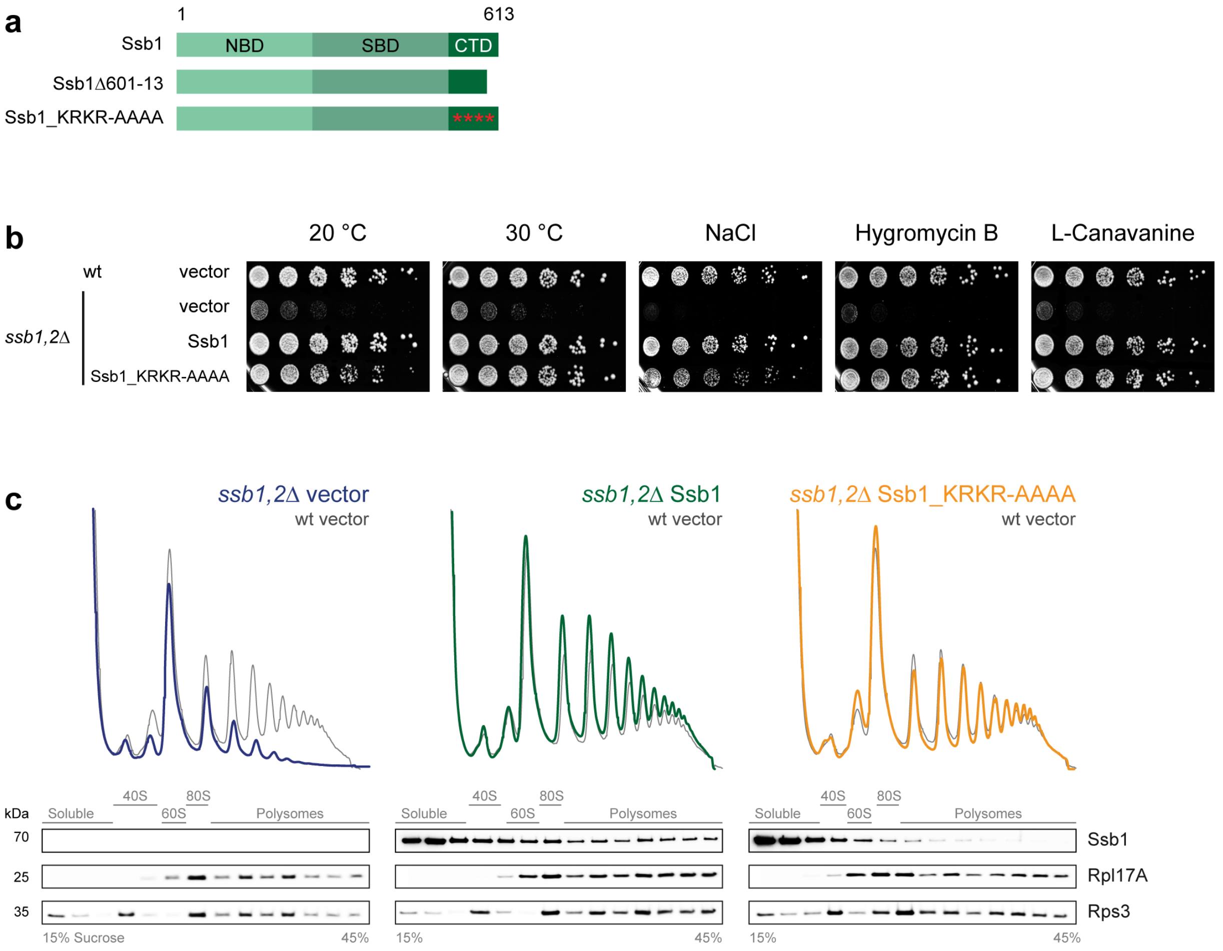

Supplementary Figure 4: Substitution of basic to neutral residues within the C-terminus of Ssb1 negatively influences ribosomal interaction. a) Schematic overview of Ssb domains and the constructs used. Ssb1Δ601-13 lacks 13 C-terminal residues; red asterisks mark point mutations (Ssb1_KR603AA-K608A-R613A). **b)** Growth analysis of wt and *ssb1,2Δ* cells transformed with either empty vector or different Ssb1 constructs. Exponentially growing cells were adjusted to OD_{600} = 0.4 and spotted in fivefold serial dilutions onto SC-URA plates (that may contain certain additives) which were incubated at 30 °C for two days or at 20 °C for five days. **c)** For polysome profiling wt cells transformed with empty vector (grey) or *ssb1,2Δ* cells transformed with either empty vector (blue) or different Ssb1 constructs (green, orange) were grown in SC-URA media to early exponential phase. Lysates were adjusted and 18 A_{260} units on top of a linear 15-45 % (w/v) sucrose gradient were ultracentrifuged followed by gradient fractionation from top to bottom and OD_{254} monitoring (top). Fractions were analyzed via immunoblotting (bottom). The wt profile (grey) at the background of each profile served as control.

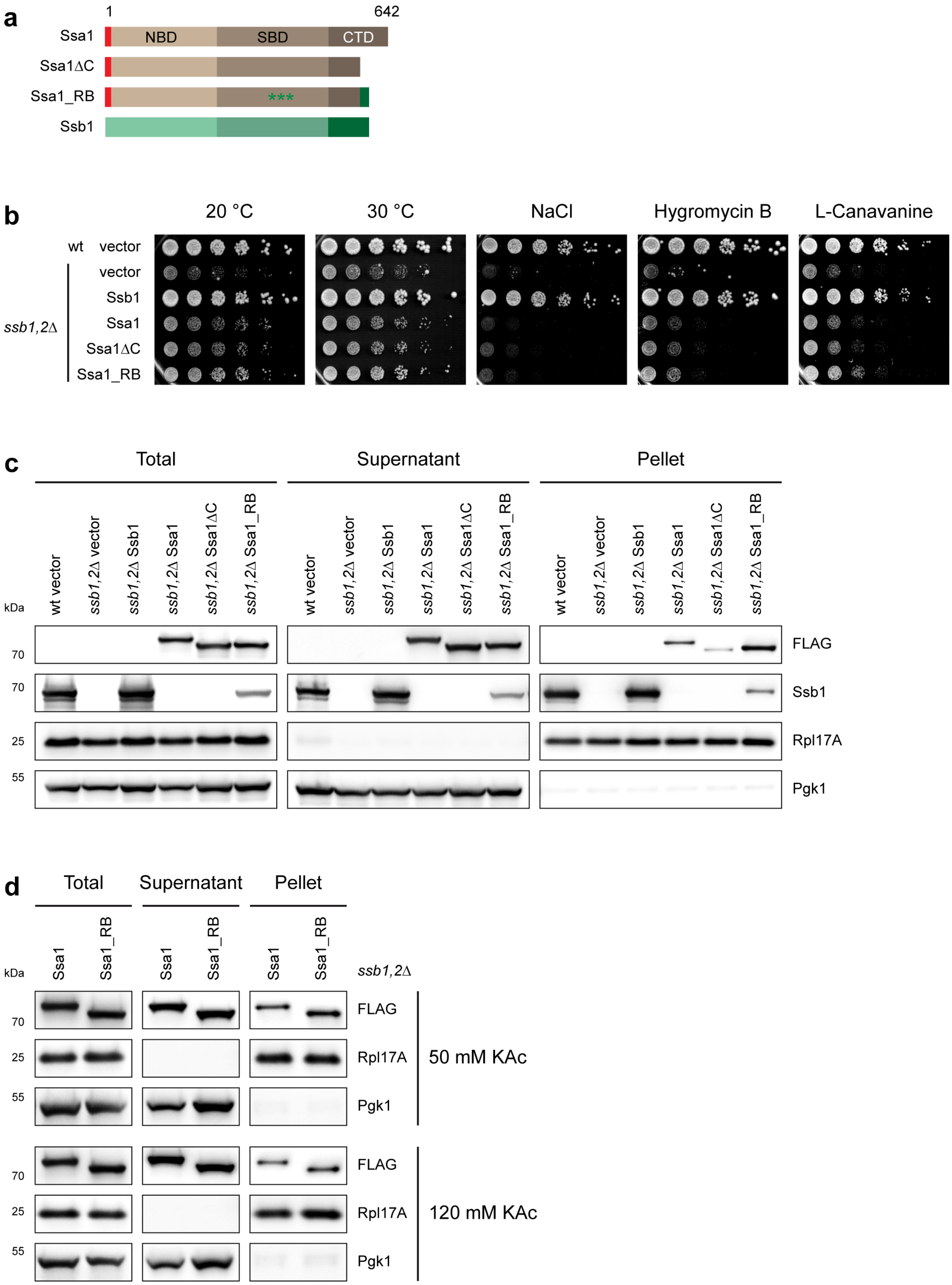

Supplementary Figure 5: Insertion of Ssb ribosome-binding sites into Ssa1 enhances targeting of this Hsp70 to ribosomes. a) Schematic overview of the constructs used. All Ssa1 versions are tagged with an N-terminal FLAG (red); Ssa1ΔC and Ssa1_RB (ribosome binding) lack residues 593-642; Ssa1_RB is C-terminally fused to Ssb1 residues 601-13 and its KSE421 region is substituted by the Ssb1 KRR-motif (asterisks). **b)** Growth analysis of

different Ssa1 constructs. Wt or *ssb1,2Δ* cells transformed with either empty vector, Ssb1 or different Ssa1 versions were grown to exponential phase, adjusted to OD_{600} = 0.4 and spotted in fivefold serial dilutions onto SC-URA plates (that may contain certain additives) which were incubated at 30 °C for two days or at 20 °C for five days. **c)** Ribosome sedimentation assay to investigate ribosome binding of different Ssa1 mutants. Cells were grown to exponential growth phase, lysates were adjusted to same A_{260} levels and 1.5 A_{260} units were loaded onto a 25 % (w/v) sucrose cushion followed by ultracentrifugation. Equal amounts of adjusted total, supernatant and ribosomal pellet fractions were analyzed via SDS-PAGE followed by Western blotting (note that the Ssb-specific antibody detects the Ssb1 residues in Ssa1_RB). **d)** Ribosome sedimentation as shown in c) under different potassium acetate (KAc) concentrations.

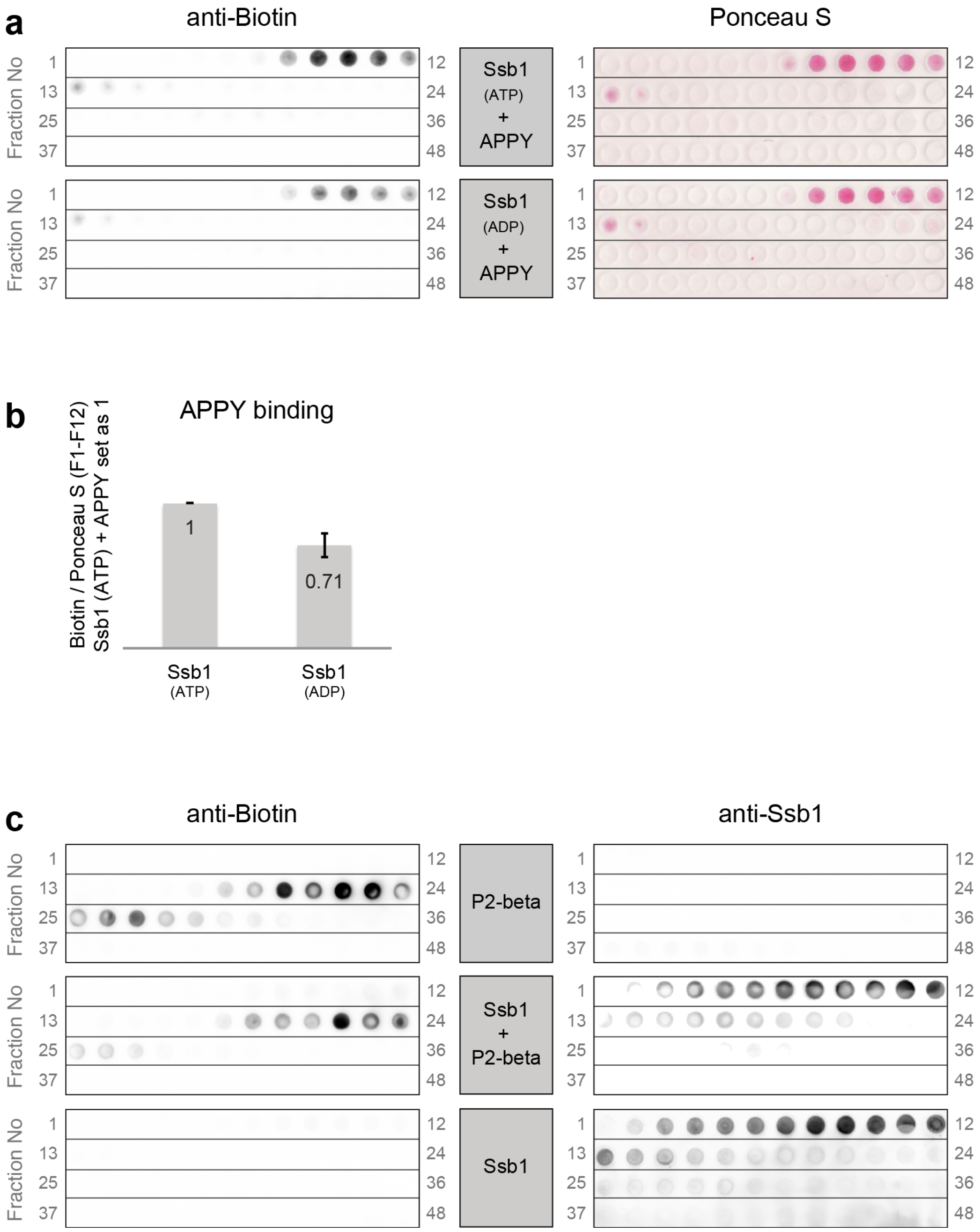

Supplementary Figure 6: The interaction of Ssb1 and APPY peptide is specific and dependent on the nucleotide state of Ssb. a) Analysis of Ssb1 and APPY-peptide interaction via gel filtration in dependency of the nucleotide state. Ssb1 was either pre-treated with ATP (top) or apyrase (bottom) followed by incubation with biotinylated APPY peptide. Upon size exclusion chromatography the eluted fractions were spotted from high to low molecular weight (grey numbers indicate fractions) onto nitrocellulose membranes followed by detection of either biotinylated APPY (via HRP-StrepTactin; left) or Ssb1 (via Ponceau S staining; right). **b)** Quantification of shifted APPY peptide in dependency of the nucleotide state of Ssb1 at the time point of peptide addition as shown in a). Biotin and Ponceau S signals from fractions 1-12 were quantified via ImageJ and the interaction of $Ssb1_{ATP}$ with APPY was set as 1 (Biotin / Ponceau S signal). Error bars represent s.e.m. of at least three independent experiments. **c)** Ssb1 does not interact with the acidic peptide P2-beta. Biotinylated P2-beta was incubated either alone (top) or together with Ssb1 protein (middle). Ssb1 alone served as control (bottom). Fractions upon size exclusion were analyzed for the presence of peptide (via StrepTactin; left) or Ssb1 protein (via Ssb1 specific antibody; right).

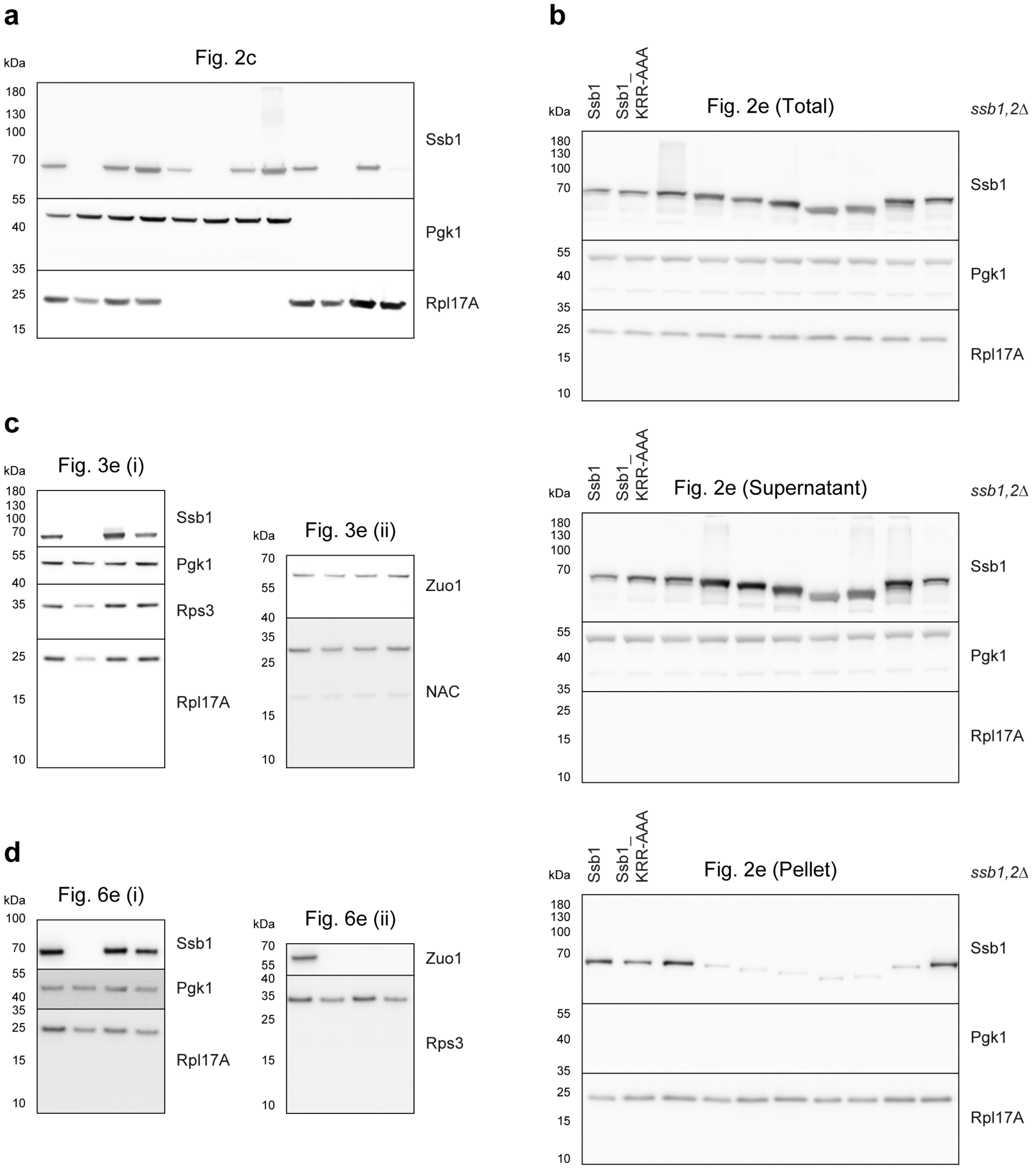

Supplementary Figure 7: Uncropped images of the most important immunoblot analyses of the main figures. a) Corresponding image of Figure 2c. **b)** Corresponding images of Figure 2e Total (top), Supernatant (middle) and Pellet (bottom). **c)** Corresponding images of Figure 3e. **d)** Corresponding images of Figure 6e.
